# Long Non-coding RNA Rhabdomyosarcoma 2-Associated Transcript Regulates Angiogenesis in Endothelial Cells

**DOI:** 10.3389/fphys.2021.729157

**Published:** 2021-10-21

**Authors:** Maha Alaqeeli, Dominique Mayaki, Sabah N. A. Hussain

**Affiliations:** ^1^Translational Research in Respiratory Diseases Program, Research Institute of the McGill University Health Centre, Department of Critical Care, McGill University Health Centre, Montréal, QC, Canada; ^2^Meakins-Christie Laboratories, Department of Medicine, McGill University, Montréal, QC, Canada

**Keywords:** angiogenesis, endothelial cells, cell signaling, non-coding RNAs, lncRNAs, cell migration, proliferation, cell differentiation

## Abstract

**Background:** Long non-coding RNAs (lncRNAs) are non-coding RNAs that have more than 200 nucleotides. They have recently emerged as important regulators of angiogenesis. To identify novel lncRNAs that may be involved in the regulation of angiogenesis, we detected the mRNA of 84 lncRNAs in human umbilical vein endothelial cells (HUVECs) exposed to hypoxia for 24h. One of these, rhabdomyosarcoma 2-associated transcript (RMST), is significantly upregulated by hypoxia. Little is known about the presence and roles of RMST in EC function.

**Objective:** The main objective of the study was to investigate the regulation of RMST in ECs and to determine its role in EC survival, proliferation, migration, and differentiation.

**Methods:** Using qPCR, basal mRNA levels of 10 RMST isoforms in HUVECs were measured. Levels were then measured in response to 24h of hypoxia, 7days of differentiation in a co-culture assay, and exposure to four different angiogenesis factors. Functional roles of RMST in EC survival, migration, and differentiation were quantified by using a loss-of-function approach (transfection with single-stranded antisense LNA GapmeRs). EC survival was measured using cell counts and crystal violet assays. Cell migration and differentiation were measured using scratch wound healing and Matrigel® differentiation assays, respectively.

**Results:** Five RMST isoforms (RMST-202, -203, -204, -206, and -207) were detected in HUVECs and human microvascular endothelial cells (HMEC-1s). Other types of vascular cells, including human aortic valve interstitial cells and human aortic smooth muscle cells, did not display this expression profile. RMST was significantly upregulated in response to 24h of hypoxia and in response to 7days of HUVEC co-culture with human lung fibroblasts. RMST was significantly downregulated by angiopoietin-2 (Ang-2), but not by VEGF, FGF-2, or angiopoietin-1 (Ang-1). Selective knockdown of RMST demonstrated that it promotes EC survival in response to serum deprivation. It is also required for VEGF- and Ang-1-induced EC survival and migration, but not for differentiation.

**Conclusion:** We conclude that RMST is expressed in human ECs and that this expression is upregulated in response to hypoxia and during differentiation into capillary-like structures. We also conclude that RMST plays important roles in EC survival and migration.

## Introduction

Angiogenesis is the formation of new blood vessels from pre-existing blood vessels. It is of paramount physiological importance to normal embryonic vascular development, adult vascular regeneration and repair, and the vascular remodeling that is associated with diseases, such as atherosclerosis, pulmonary fibrosis, and tumor growth (Carmeliet, 2003). It is therefore crucial to identify and understand the functional significance of the regulatory molecules that control angiogenic processes under normal and pathological conditions. By doing so, we increase the likelihood that novel therapies will be developed that improve normal angiogenesis or inhibit pathological angiogenesis.

Angiogenesis in endothelial cells (ECs) unfolds as a complex interplay of multiple cellular processes, including activation, proliferation, migration, and differentiation. In recent years, epigenetic regulators of gene expression known as non-coding RNAs (ncRNAs) have emerged as important modulators of angiogenesis. Based on their length, ncRNAs can be characterized as either short or long. The short version, where transcripts have less than 200 nucleotides, is known as microRNAs (miRNAs), and their roles in angiogenesis have been extensively documented over the past several years. ncRNAs that have more than 200 nucleotides are simply known as long non-coding RNAs (lncRNAs). They are transcribed by RNA polymerase II and are classified into five categories: long intergenic, bidirectional, intronic, sense, and antisense (DiStefano, 2018). lncRNAs regulate gene expression by altering chromatin, by functioning as a source of miRNAs, by “sponging” endogenous miRNAs, or by acting as scaffolds for protein complexes (Simion et al., 2019). Several, including MALAT1, TUG1, MEG3, and LINC00657, have been shown to be abundantly expressed in ECs.

Approximately 56% of the total RNA in human umbilical vein endothelial cells (HUVECS) is non-coding and 7% of that number is represented by lncRNAs (Michalik et al., 2014), suggesting that there are yet to be identified lncRNAs that may regulate angiogenesis. To identify them, we conducted pilot experiments that used RT2™ lncRNA PCR arrays to detect lncRNA expression in HUVECs exposed to 24h of hypoxia (angiogenic stimulus). Several lncRNAs were significantly upregulated, including rhabdomyosarcoma 2-associated transcript (RMST). RMST was first identified as a functional ncRNA by Chan et al. (2002). Subsequent reports indicated that it is relatively abundant in brain regions and that it plays important roles in murine brain development and neuronal differentiation (Bouchard et al., 2005; Uhde et al., 2010; Ng et al., 2013). It has also been detected in triple-negative breast cancer cells (TNBCs; Yang et al., 2016; Wang et al., 2018).

To the best of our knowledge, RMST has only been detected in ECs by Yin et al. who reported that RMST expression is significantly upregulated in human and mouse brain ECs in response to acute oxygen-glucose deprivation (OGD) and that RMST knockdown attenuates OGD-induced injury *via* regulation of the miR-204-5p/vascular cell adhesion molecule 1 (VCAM1) axis (Yin et al., 2021). Whether RMST plays a role in other EC functions, such as angiogenesis, remains unknown. Accordingly, the main aims of this study are to investigate the regulation of RMST expression in ECs and to determine its role in mediating EC survival, proliferation, migration, and differentiation.

## Materials and Methods

### Materials

The study was approved by the ethics committee of the McGill University Health Centre. Human umbilical vein endothelial cells (HUVECS) and primary human aortic smooth muscles (HASMCs) were purchased from Thermo Fisher Scientific (Frederick, MD). Green fluorescent protein (GFP)-expressing HUVECs that were generated by infecting HUVECs pooled from multiple donors with lentiviruses expressing GFP were purchased from Angio-Proteomie (Boston, MA). Immortalized human dermal microvascular endothelial cells (HMEC-1) were provided by Dr. Anie Philip (McGill University). Primary human lung fibroblasts were provided by Dr. Carolyn Baglole (McGill University). Primary human aortic valve interstitial cells (HAVICs) were provided by Dr. Adel Schwertani (McGill University). Recombinant human angiopoietin-1 (Ang-1), angiopoietin-2 (Ang-2), vascular endothelial growth factor (VEGF), and fibroblast growth factor 2 (FGF-2) were purchased from PeproTech (Rocky Hill, NJ). RMST-specific antisense LNA GapmeRs and a control GapmeR were purchased from Exiqon (Woburn, MA). Lipofectamine RNAiMAX reagent and a SuperScript™ First-Strand synthesis system for RT-PCR were obtained from Invitrogen (Burlington, ON).

### Cell Culture

HUVECs and GFP-HUVECs were used between passages 4 to 7. HUVECs, GFP-HUVECs, and HMEC-1 cells were grown in complete MCDB131® medium (Life Technologies, Rockville, MD) supplemented with 20% fetal bovine serum (FBS), endothelial cell growth supplement, 2mmol/l glutamine, heparin, and gentamicin and incubated at 37°C and 5% CO_2_. Primary human lung fibroblasts were cultured in MEM medium (Life Technologies, Rockville, MD) supplemented with 10% FBS and antibiotics. HAVICs, generated as previously described (Yu et al., 2017), were used at passages 3–5 and cultured in DMEM medium supplemented with 10% FBS, glutamine, and antibiotics. HASMCs were cultured in smooth muscle cell growth medium (SmGM™ 2; Lonza, Basel, Switzerland) supplemented with 5% FBS, insulin, FGF-B, gentamycin, amphotericin, and epithelial growth factor (EGF).

### RMST Expression

In mammalian cells, 10 RMST isoforms have been identified, ranging from the relatively short transcript RMST-207 (60 nucleotides) to the relatively long transcript RMST-209 (3,269 nucleotides; Table 1, Ensembl.org). Full sequences of isoforms are listed in Supplementary Table 1. Relative expressions of RMST isoforms were measured in two endothelial cell types (HUVECs and HMEC-1s), vascular smooth muscle cells (HASMCs), and vascular interstitial cells (HAVICs). Total RNA was extracted using a PureLink™ RNA Mini Kit (Invitrogen) according to the manufacturer’s protocol. Following spin-column extraction of nucleic acids (Invitrogen), reverse transcription was performed using Superscript™ II Reverse Transcriptase. Transcripts were detected using a Real-Time PCR System 7500 (Applied Biosystems, Foster City, CA) and SYBR™ Green PCR Master Mix (Thermo Fisher Scientific) with specific primers (Supplementary Table 1). β-actin, glyceraldehyde 3 phosphate dehydrogenase (GAPDH), and 18S ribosomal RNA expression were used as controls. Results were analyzed using the ΔΔC_T_ method in which ΔC_T_ is the difference between the gene of interest and the geometric means of the housekeeping genes, and ΔΔC_T_ is average ΔΔC_T_ of the control group minus ΔC_T_ of the test gene. RMST isoform expressions in various cells were also shown as log values of ΔC_T_. All qPCR experiments were performed in triplicate.

**Table 1:** Human RMST isoforms.

Isoform name	No. of exons	No. of nucleotides	Type
RMST-201	14	2,598	lncRNA
RMST-202	9	2099	lncRNA
RMST-203	1	687	retained intron
RMST-204	2	755	retained intron
RMST-205	7	1,047	lncRNA
RMST-206	2	656	retained intron
RMST-207	1	60	miscellaneous RNA
RMST-208	14	2099	lncRNA
RMST-209	16	3,269	lncRNA
RMST-210	14	2,844	lncRNA

### Subcellular Localization of RMST

A Norgen Biotek Cytoplasmic and Nuclear RNA Purification Kit (Thorold, ON) was used according to the manufacturer’s protocol to isolate and purify total cytoplasmic and nuclear RNA from HUVECs cultured in complete medium. Purified RNA was reverse-transcribed to cDNA, and gene expressions of RMST isoforms were measured using qPCR.

### Regulation of RMST Expression During Cell Differentiation

A co-culture angiogenesis assay (Hetheridge et al., 2011) was used to assess RMST expression during EC differentiation. In brief, GFP-HUVECs were cultured in Lonza EGM™-2 BulletKit™ medium supplemented with 2% FBS, SingleQuots™ supplements and gentamicin. Human lung fibroblasts were grown on 10-cm culture plates and maintained in DMEM medium supplemented with 10% FBS. When fibroblasts reached full confluence, 500,000 GFP-HUVECs were added on top of them, and the medium was changed to Lonza EGM™-2 BulletKit™ medium. Medium was changed every other day. After 7days of co-culture, cells were detached with trypsin and the GFP-HUVECs were sorted out using fluorescence-activated cell sorting (FACS) and a BD™ FACSAria Fusion cell sorter located at the Immunophenotyping Platform of the Research Institute of the McGill University Health Centre. As a control, GFP-HUVEC/fibroblast co-culture was maintained for 1day prior to cell sorting. Total RNA was extracted from sorted GFP-HUVECs, and gene expressions of RMST isoforms were measured using qPCR as described above.

### Regulation of RMST Expression by Acute Hypoxia and Angiogenesis Factors

For hypoxia experiments, HUVECs were cultured in an incubator chamber (Billups-Rothenberg, Del Mar, CA) in complete MCDB131 medium under normoxic or hypoxic (5% CO_2_ and 95% N_2_) conditions for 24h. Total RNA was extracted, and gene expressions of RMST isoforms were measured using qPCR as described above. For angiogenic factor experiments, HUVECs were maintained in basic MCDB131 medium (2% FBS, no growth supplement) for 6h and then transferred to basic medium containing aliquots of phosphate-buffered saline (PBS, control), vascular endothelial cell growth factor (VEGF, 40ng/ml), angiopoietin-1 (Ang-1, 300ng/ml), angiopoietin-2 (Ang-2, 300ng/ml), or fibroblast growth factor 2 (FGF-2, 10ng/ml). Cells were collected 24h later, and RMST-202 expression was measured as described above.

### Transection With LNA GapmeRs

LNA GapmeRs are single-stranded antisense oligonucleotides that catalyze RNase H-dependent degradation of complementary RNA targets. They were used to determine the functional importance of RMST in *in vitro* angiogenesis. Using LipofectamineTM RNAiMAX (Invitrogen) according to the manufacturer’s protocol, HUVECs were transfected with 6.25, 12.5, 25, or 50nM of LNA GapmeRs (Exiqon Inc.). GapmeRs were scrambled (control) or selectively directed against RMST-202 and RMST-206 (most abundant RMST isoforms in ECs). All experiments were performed 48h post-transfection. Knockdown was verified with qPCR.

### EC Cell Survival

Cell counting and crystal violet assays were used to determine the functional roles of RMST in EC survival. For cell counts, equal numbers of control or RMST GapmeR-transfected HUVECs (30,000 cells per cm2) were maintained for 24 or 48h in complete or basic (2% FBS) MCDB131 medium. Cell were counted using a hemocytometer. For crystal violet assays, equal numbers of GapmeR-transfected HUVECs (30,000 cells per cm^2^) were maintained for 24h in complete or basic (2% FBS) MCDB131 medium containing aliquots of PBS, VEGF (40ng/ml), or Ang-1 (300ng/ml). Cell survival was measured as previously described (Šliwka et al., 2016). Briefly, at the end of the experimental period, medium was removed, 20% methanol was added, and cells were maintained for 15min at room temperature. Crystal violet solution (0.5%) was added, and cells were maintained for a further 15min at room temperature. Plates were then washed and dried, and 1% SDS was added to solubilize the cells. Absorbance at 590 was measured with an Infinite 200 PRO multimode plate reader (Tecan, Männedorf, Switzerland).

### EC Proliferation

GapmeR-transfected HUVECs were plated in 96-well plates (15,000 cells per well) and maintained in complete MCDB131 medium. Bromodeoxyuridine (BrdU; Millipore, Etobicoke, ON) was added 1h after plating. Cells were collected 24 and 48h later. BrdU absorbance was measured with an Infinite 200 PRO multimode plate reader (Tecan).

### Cell Migration Assays

EC migration was measured using a scratch (wound) healing assay as previously described (Abdel-Malak et al., 2008). GapmeR-transfected HUVECs were grown as monolayers and then wounded with a 200-μl pipette tip. Cells were maintained in basic medium containing PBS or Ang-1 (300ng/ml) for 8h. Wounded areas were imaged using an Olympus inverted microscope and quantified using Image-Pro Plus™ software (Media Cybernetics, Bethesda, MD). Values are reported as % wound healing and calculated according to the following formula:


$$\begin{array}{l} \%\mathrm{wound healing}=\left[1-\left(\mathrm{wound area}\;\mathrm{at}\;t8/\mathrm{wound area}\;\mathrm{at}\;\mathrm{t0}\right)\right]\\ \;\;\times 100 \end{array}$$

where t8 is the time (8h) over which cells were maintained in media and t0 is the time immediately following wounding.

### EC Differentiation

GapmeR-transfected HUVECs were seeded onto 96-well plates pre-coated with growth factor-reduced Matrigel™ (BD Biosciences, Bedford, MA) at a density of 5×10^4^ cells per well. Cells were maintained in basic MCDB131 medium for 6h. Whole-well images were captured using an Olympus inverted microscope (40X) and analyzed using Image-Pro Plus™ software. Angiogenic tube formation was determined by counting the number of tube branches and measuring total tube length in the analyzed areas using the angiogenesis analyzer ImageJ, as previously described (Sanchez et al., 2019).

### RMST Regulation of Angiogenesis and the Cell Cycle

To investigate the mechanisms through which RMST regulates *in vitro* angiogenesis, the effect of RMST knockdown on VEGF, the Ang-1 pathway, and the cell cycle were measured. Using a PureLink™ RNA Mini Kit, total RNA was extracted from cells 48h post-transfection with scrambled or RMST GapmeRs. Reverse transcription was performed using Superscript™ II Reverse Transcriptase. Transcripts were detected using a Real-Time PCR System 7,500, SYBR™ Green PCR Master Mix with primers specific to VEGF, VEGF receptor 1 (VEGFR1), VEGF receptor 2 (VEGFR2), VEGF-C, Ang-1, Ang-2, Tie-2, cyclin D1, cyclin A2, cyclin E1, cyclin-dependent kinase 1 (CDK1), and cyclin-dependent kinase 2 (CDK2) (Supplementary Table 1). β-actin, glyceraldehyde 3 phosphate dehydrogenase (GAPDH), and 18S ribosomal RNA expression were used as controls. Normalized gene expression was expressed in log values.

### Data Analysis

Data were expressed as means±standard error of the means (SE). Statistical significance was determined by one-way analysis of variance. *p* values of less than 0.05 were considered statistically significant.

## Results

### RMST Expression in ECs

Supplementary Table 2 shows pilot experiment results that indicate that the expressions of six lncRNAs (CCAT2, GAS5, H19, HOTAIR, MALAT1, and RMST) were significantly upregulated in HUVECs exposed to 24h of hypoxia (angiogenic stimulus). The relative induction of RMST was higher than the other five. Expressions were detected using an RT2™ lncRNA PCR Array Human lncFinder™ kit (Qiagen Inc.).

**Figure 1:**
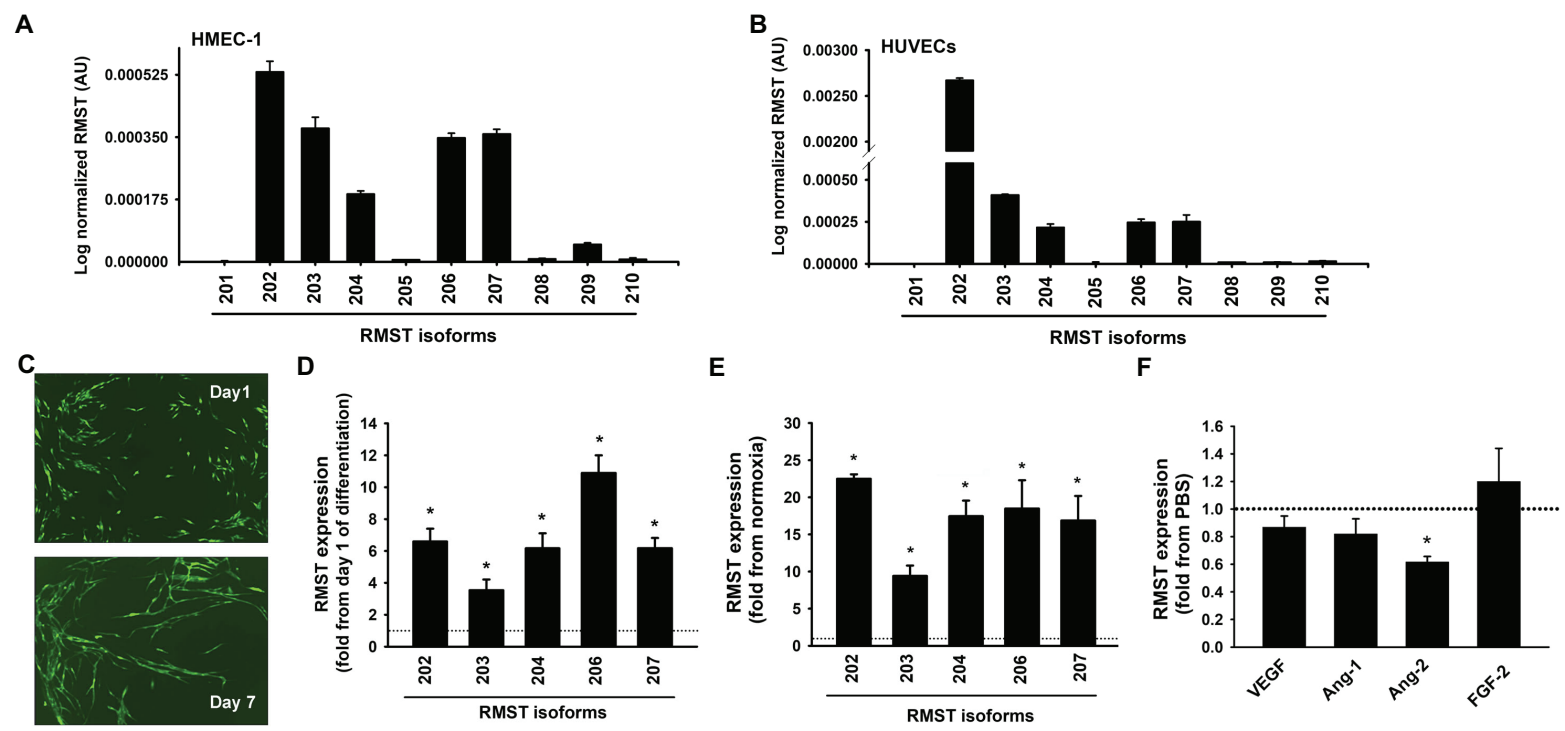
RMST expression in human ECs. (A,B) Log normalized mRNA expressions of various RMST isoforms in human umbilical vein endothelial cells (HUVECs; **A**) and human microvascular endothelial cells (HMECs; **B**). Values are means±SEM, expressed as arbitrary units. *N*=6 per isoform. **(C)** Representative angiogenesis assay images in which GFP-HUVECs were co-cultured with human lung fibroblasts for 1 (top) and 7 (bottom) days. Note differentiation of cells into tube-like structures. **(D)** mRNA expressions of 5 RMST isoforms in GFP-HUVECs after 7days of co-culture with human lung fibroblasts. Values are means±SEM, expressed as fold from day 1 of co-culture values. *p<0.05, compared to day 1. *N*=5. **(E)** mRNA expressions of 5 RMST isoforms in HUVECs in response to 24h of hypoxia (5% CO_2_ and 95% N_2_). Values are means±SEM, expressed as fold from normoxic values. *p<0.05, compared to normoxia. *N*=8. **(F)** mRNA expressions of RMST-202 in HUVECs maintained in basic medium then exposed to PBS (control), VEGF (40ng/ml), Ang-1 (300ng/ml), Ang-2 (300ng/ml), and FGF-2 (10ng/ml) for 24h. Values are means±SEM, expressed as fold from PBS. *p<0.05, compared to PBS. *N*=6.

Five RMST isoforms (RMST-202, -203, -204, -206, and -207) were detected in HUVECs maintained in complete MCDB131 medium, with RMST-202 having the highest relative expression compared to the other isoforms (Figure 1A). Six RMS isoforms (RMST-202, -203, -204, -206, -207, and -209) were detected in HMEC-1s (Figure 1B). To determine whether this pattern of expression is specific to ECs, RMST expression was also measured in normoxic primary human aortic smooth muscles (HASMCs) and primary human aortic valve interstitial cells (HAVICs). RMST-206, -207, -209, and -210 were detected in HASMCs, while RMST-202, -206, -207, and -209 were detected in HAVICs. In both cell types, RMST-209 was the most abundantly expressed isoform (Supplementary Figure 1). These results suggest that ECs exhibit a unique pattern of RMST expression as compared to other vascular cells. Separation of cellular contents into nuclear and cytosolic fractions revealed that all detectable RMST isoforms in HUVECs were localized to the nucleus (Supplementary Figure 2). To determine whether RMST expression changes during EC differentiation, HUVECs were co-cultured for 7days with human lung fibroblasts. In this model of angiogenesis, HUVECs differentiate into tube-like structures resembling *in-vivo* capillaries (Figure 1C). Differentiation was associated with significant upregulation of all detectable RSMT isoforms (Figure 1D). Acute hypoxia, another known stimulus of angiogenesis, also upregulated RMST expressions (Figure 1E). To determine the effects of various angiogenic factors on RMST expression in ECs, RMST-202 expression was measured in HUVECs exposed to VEGF, Ang-1, Ang-2, and FGF-2 for 24h. Ang-2 downregulated RMST-202. VEGF, Ang-1, and FGF-2 had no effect on RMST-202 (Figure 1F).

### Transfection With LNA GapmeRs

**Figure 2:**
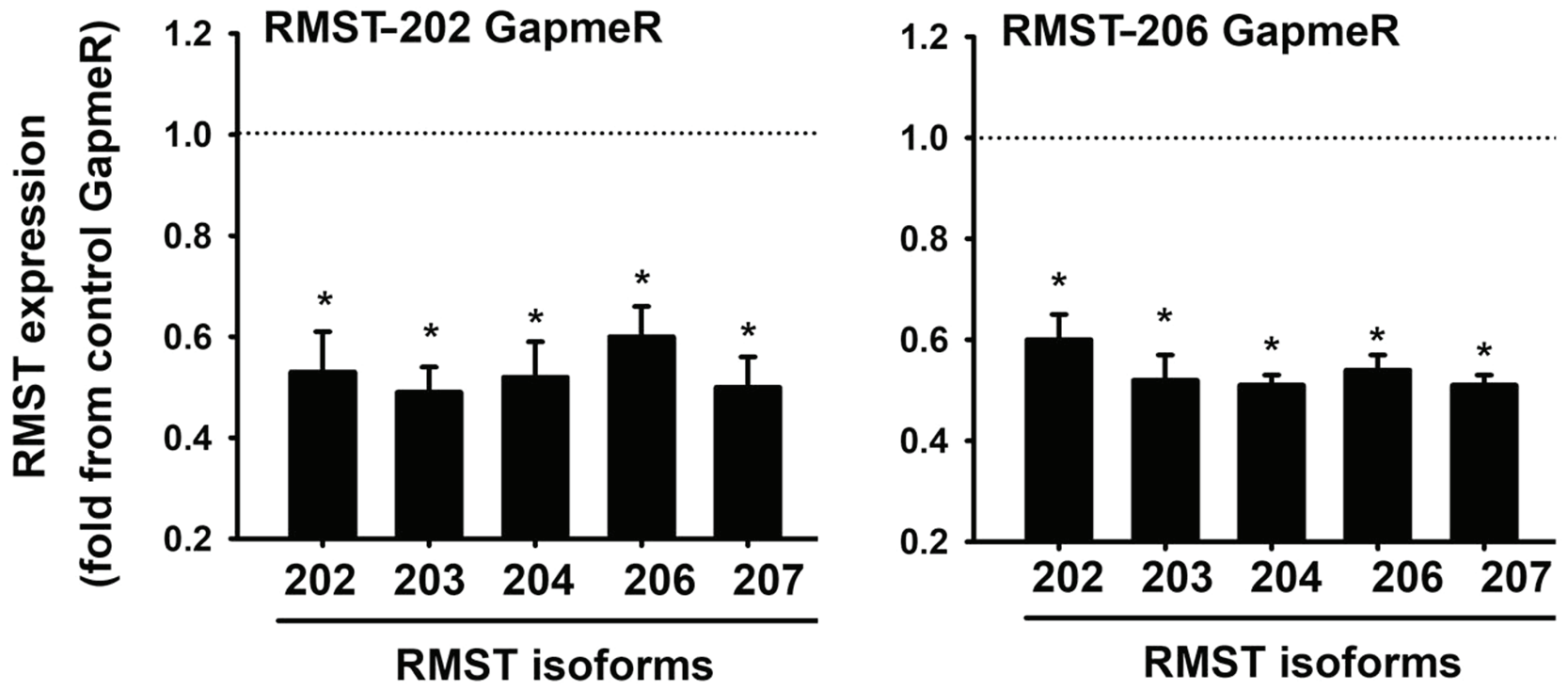
LNA GapmeR knockdown of RMST in HUVECs. mRNA expressions of 5 RMST isoforms in HUVECs 48h post-transfection with 12.5nM of scrambled (control) GapmeR, 12.5nM of RMST-202 GapmeR (**left**), or 6.25nM of RMST-206 GapmeR (**right**). Values are means±SEM, expressed as fold from control GapmeR. *p<0.05, compared to control GapmeR. *N*=8.

Since RMST-202 and -206 are the most abundantly expressed isoforms in HUVECs, we used a loss-of-function approach to selectively knock them down using selective GapmeRs. First, we identified the concentration of GapmeR that elicited the most significant downregulation of RMST. The strongest downregulation of RMST-202 was achieved with 12.5nM, while significant downregulation of RMST-206 was achieved with 6.25nM (Supplementary Figure 3). However, neither the RMST-202 GapmeR nor the RMST-206 GapmeR were specific since all other isoforms were also downregulated by them (Figure 2). We concluded from this that it was not possible to downregulate a specific isoform without affecting the expressions of others. Based on this, the RMST-202 GapmeR was used in all subsequent experiments that were designed to determine the functional roles of RMST in cultured ECs.

### RMST Regulation of EC Survival

**Figure 3:**
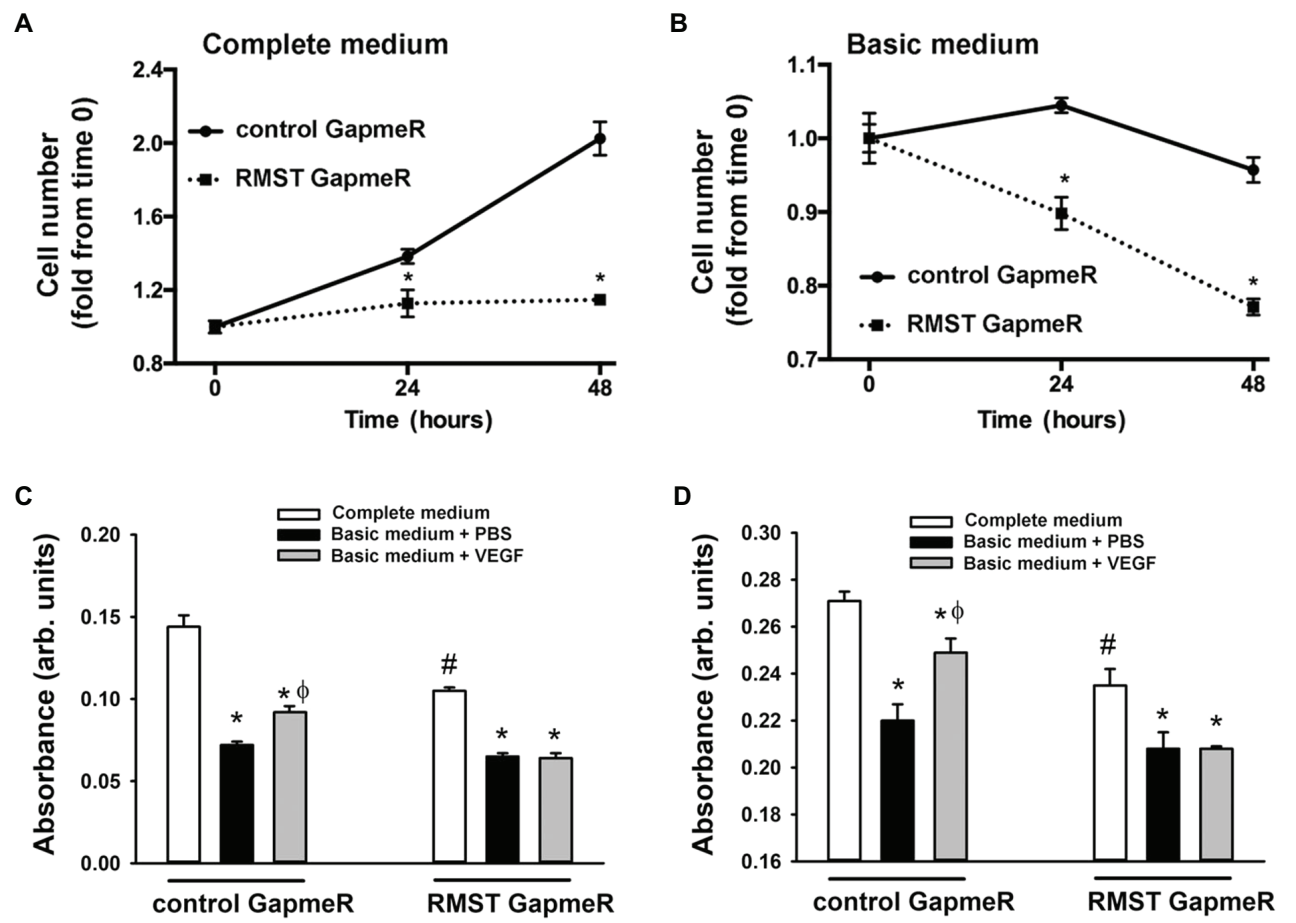
RMST regulation of endothelial cell survival. **(A,B)** Hemocytometer cell counts of equal numbers of control and RMST GapmeR-transfected HUVECs maintained for 24 and 48h in complete MCDB131medium (left) or basic MCDB131 medium (right). Values are means±SEM, expressed as fold from time 0. *p<0.05, compared to control GapmeR. *N*=6. **(C,D)** Hemocytometer cell counts of equal numbers of control and RMST GapmeR-transfected HUVECs maintained for 24h in complete medium **(C,D)**, basic medium containing PBS (C&D), basic medium containing VEGF **(C)**, or basic medium containing Ang-1 **(D)**. Cell survival determined by crystal violet staining. Values are means±SEM, expressed in arbitrary units. *p<0.05, compared to complete medium. ^ϕ^*p*<0.05, compared to PBS. ^#^*p*<0.0, compared to control GapmeR-transfected cells maintained in complete medium.

Two assays were used to determine the functional roles of RMST in EC survival. In the first, a cell counting assay was used to determine the role of RMST in EC survival in response to acute serum starvation (Figures 3A,B). Equal numbers of control or RMST GapmeR-transfected HUVECs were maintained for 24 or 48h in complete or basic MCDB131 medium. In both media, counts of cells transfected with the control GapmeR were significantly higher than those of cells transfected with the RMST-202 GapmeR, suggesting that RMST is required for EC survival (Figures 3A,B). In the second assay, crystal violet stain was used to determine the role RMST plays in the pro-survival effects of VEGF and Ang-1 (Figures 3C,D). Cells transfected with control or RMST GapmeRs were maintained for 24h in complete or basic medium containing aliquots of PBS (control), VEGF, or Ang-1. Crystal violet absorbance was lower for serum-deprived control GapmeR-transfected cells as compared to those maintained in complete medium. When serum-deprived control GapmeR-transfected cells were exposed to VEGF and Ang-1, absorbance increased relative to basic medium alone, but remained lower than what was measured in complete medium (Figures 3C,D). This demonstrates that VEGF and Ang-1 promote EC survival. Neither VEGF nor Ang-1 had any effect on absorbance when measured in serum-deprived RMST-202 GapmeR-transfected cells, indicating that the pro-survival effects of VEGF and Ang-1 require RMST.

### RMST Regulation of EC Proliferation and Migration

**Figure 4:**
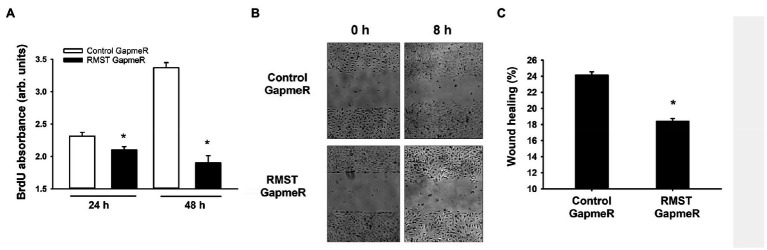
RMST regulation of endothelial cell proliferation and migration. **(A)** BrdU absorbance of equal numbers of control or RMST GapmeR-transfected HUVECs maintained in complete medium containing BrdU for 24 and 48h. Values are means±SEM, expressed in arbitrary units. *p<0.05, compared to control GapmeR. **(B)** Representative micrographs of scratch wound healing at times 0 and 8h in control or RMST GapmeR-transfected HUVECs maintained in basic medium. **(C)** Percent wound healing in control or RMST GapmeR-transfected HUVECs maintained in basic medium. Values are means±SEM. *p<0.05, compared to control GapmeR. *N*=8.

To investigate whether RMST regulates EC proliferation, BrdU incorporation was measured in control and RMST GapmeR-transfected HUVECs maintained in complete medium for 24 or 48h. BrdU incorporation was lower in RMST GapmeR-transfected cells relative to control GapmeR-transfected cells, indicating that RMST promotes EC proliferation (Figure 4A).

The rate of EC wound healing in control GapmeR-transfected HUVECs was approximately 24%. It was significantly lower in RMST GapmeR-transfected cells, indicating that endogenous RMST is required for EC migration (Figures 4B,C).

### RMST Regulation of EC Differentiation

**Figure 5:**
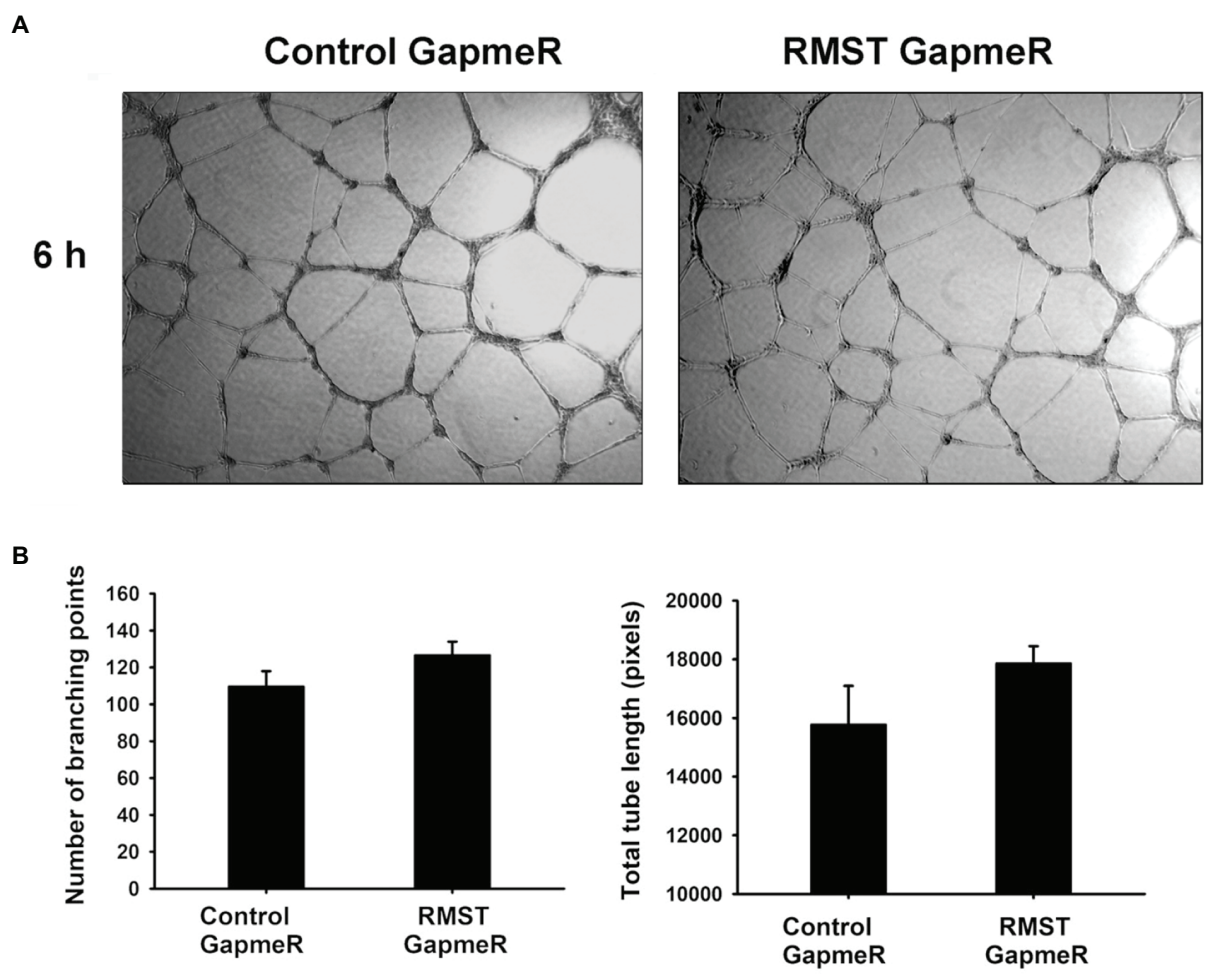
RMST regulation of endothelial cell differentiation. **(A)** Representative micrographs of control or RMST GapmeR-transfected HUVECs maintained in basic medium for 6h on growth factor-reduced Matrigel®-coated plates. **(B)** Number of branching points (left) and total tube length (right) of control or RMST GapmeR-transfected HUVECs maintained in basic medium for 6h on growth factor-reduced Matrigel®-coated plates. Values are means±SEM. *N*=6.

EC tube formation assays were used to determine the functional role of RMST in acute EC differentiation (6h). Total tube length and number of branching points did not change in RMST GapmeR-transfected HUVECs compared to control GapmeR-transfected cells, suggesting that endogenous RMST does not play a significant role in acute EC differentiation (Figure 5).

### RMST Regulation of Angiogenesis and the Cell Cycle

**Figure 6:**
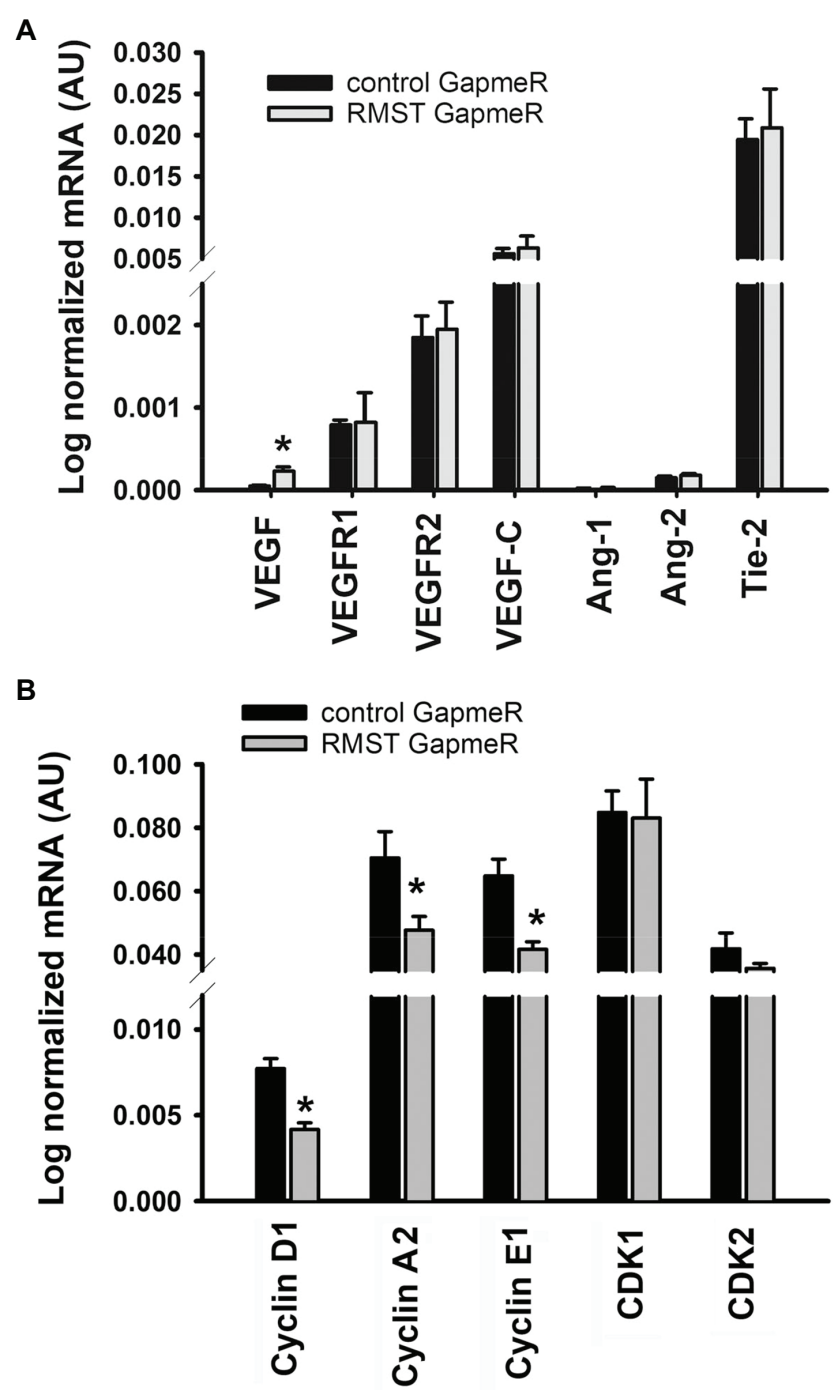
RMST regulation of angiogenesis and the cell cycle. mRNA expressions of VEGF, VEGFR1, VEGFR2, VEGF-C, Ang-1, Ang-2, Tie-2 (panel **A**), cyclins D1, A2, and E1, and CDK1 and CDK (panel **B**) in control or RMST GapmeR-transfected HUVECs. Total RNA was extracted 48h post-transfection. Values are means±SEM. *p<0.05, compared to control GapmeR. *N*=6.

Figure 6A illustrates the effects of RMST knockdown on RNA expressions of genes related to two important angiogenesis pathways (VEGF and Ang-1). Relative to control GapmeR-transfected HUVECs, VEGF expression was significantly upregulated in RMST GapmeR-transfected cells. VEGFR1, VEGFR2, VEGF-C, Ang-1, Ang-2, and Tie-2 expressions remained unchanged. Figure 6B illustrates the effects of RMST knockdown on RNA expressions of genes related to the cell cycle. Cyclin D1, E1, and A2 were significantly downregulated, while CDK1 and CDK2 did not change.

## Discussion

In this study, we characterized expression profiles and regulatory roles of the lncRNA RMST in relation to angiogenic responses of ECs. Our study demonstrates for the first time that: 1) several RMST isoforms are expressed in ECs and they are localized to the nucleus; 2) endothelial RMST expression is significantly upregulated during differentiation of ECs into capillary-like structures and in response to acute hypoxia; 3) exposure to Ang-2 but not VEGF, Ang-1, or FGF2 downregulates RMST expression in ECs; 4) RSMT plays a significant role in the pro-survival effects of VEGF and Ang-1; and 5) RMST promotes EC survival, migration, and proliferation.

### RMST Expression

Until recently, the importance of lncRNAs to the regulation of angiogenesis was largely unknown. However, several novel lncRNAs, including MANTIS (Leisegang et al., 2017), H19 (Jiang et al., 2016), MEG3 (Qiu et al., 2016), MIAT (Yan et al., 2015), PUNISHER (Kurian et al., 2015), SENCR (Yan et al., 2015), Tie-1 AS (Li et al., 2010), STEEL (Man et al., 2018), and LINC00323-3 (Fiedler et al., 2015), have now been recognized as important regulators of angiogenesis. In this study, we investigate the regulatory environment of RMST as it relates to angiogenic responses of ECs. We also explore its importance to the regulation of cell survival, proliferation, migration, and differentiation.

Alternative splicing is post-transcriptional processing of precursor mRNA to generate multiple isoforms from a single gene. It occurs either in cis or in trans configuration. In cis-splicing, different exons originating from one precursor pre-mRNA are joined. In trans-splicing, exons from two or more pre-mRNAs originating from the same gene (intragenic trans-splicing) or two or more different genes (intergenic trans-splicing) are joined. The overall function of alternative splicing is to allow for the synthesis of mRNA isoforms that may have varying cellular properties or functions.

While RMST transcripts have been detected in the normal brain and various cancer cells, it should be noted that most published studies regarding the functional roles that RMST plays in cells have been performed on the brain and neurons. For example, it has been demonstrated that RMST is abundantly expressed in midbrain dopaminergic neuronal precursors and is co-expressed with the midbrain transcription factor Lmx1a (Uhde et al., 2010). It has also been detected in human embryonic stem cells (Ng et al., 2012; Wu et al., 2014) and in cancer cells, where several studies (Chan et al., 2002; Liu et al., 2020) have located one or more isoforms in rhabdomyosarcomas, neuroblastomas, osteosarcomas, lymphomas, astrocytomas, and colon cancer. In triple-negative breast cancer tissue, relatively low levels of RMST correlate with poor patient survival, a finding that has led investigators to suggest that RMST may function as a tumor suppressor (Yang et al., 2016).

We report here for the first time that several RMST isoforms are expressed in primary human ECs. RMST-202, -203, -204, -206, -207, and -209 were detected in both HUVECs and HMEC-1s (Figure 1). RMST-202 is the most abundant isoform. With respect to size, isoforms 201, 202, 208, 209, and 210 are relatively long, possessing 9 to 16 exons, while isoforms 203, 204, 205, 206, and 207 are relatively short, possessing between 1 to 7 exons (Table 1). We also found that the RMST isoform expression profile in ECs is different from two other vascular cell types (HASMs and HAVICs), which primarily express RMST-209 and -210 (Supplementary Figure 1). These results indicate that alternative splicing of RMST pre-mRNA is cell-specific and suggest that different RMST isoforms may serve different cellular functions in different vascular cells.

Intracellular localization of RMST also differs in various tissues. In U251 glioblastoma cells, RMST is present in the mitochondria where it inhibits the autophagy pathway (Liu et al., 2020), and in neurogenic progenitor cells, it is present in the nucleus (Ng et al., 2013). In ECs, we found that RMST isoforms are localized to the nucleus (Supplementary Figure 2). We also found that RMST expression is upregulated during EC differentiation into capillary-like tube structures and in response to acute hypoxia (Figure 2). These results are qualitatively similar to those described in human embryonic stem cells as they undergo differentiation to neurons (Ng et al., 2012) and in hippocampal neurons exposed to acute hypoxia (Hou and Cheng, 2018).

Little information is available regarding precisely which transcription factors regulate RMST expression. In hindbrain cells, RMST expression is positively regulated by PAX2 (Bouchard et al., 2005), while in neuronal progenitor cells, RMST expression is negatively regulated by REST, a transcription suppressor (Ng et al., 2013). Whether they are involved in the regulation of endothelial RMST expression remains to be determined.

### Functions of RMST

As indicated above, much of the information relating to the functional roles of RMST is derived from experiments performed on the brain and neurons. We know that RMST is required for neuronal differentiation of embryonic stem cells (Ng et al., 2012) and that RMST knockdown in glioblastoma cells significantly decreases cell viability, proliferation, migration, and invasion and its overexpression elicits the opposite (Liu et al., 2020). It has also been demonstrated that RMST promotes oxygen-glucose deprivation (OGD)-induced microglial M1 polarization and neuronal apoptosis and that RMST silencing protects against OGD-induced neuron injury and middle cerebral artery occlusion (MCAO)-induced brain injury (Hou and Cheng, 2018; Sun et al., 2019; Cheng et al., 2020).

To the best of our knowledge, there is only one study that has addressed the functional roles of RMST in ECs. Recently, Yin and colleagues detected RMST in human and murine brain ECs and reported that it contributes to OGD-induced EC injury *via* upregulation of VCAM1 (Yin et al., 2021). RMST selectively binds to miR-204-5p to remove its inhibitory effect on VCAM1 expression. We report for the first time that knockdown of RMST eliminates the pro-survival effects of VEGF and Ang-1 and decreases proliferation and migration, but exerts no effect on acute differentiation (6h). To investigate the mechanisms through which RMST knockdown elicited these effects, we measured the effects of knockdown on the expressions of two important angiogenesis factors, VEGF, and Ang-1 and on several genes involved in the regulation of the cell cycle. RMST knockdown upregulated VEGF expression but had no effect on the expressions of VEGFR1, VEGFR2, VEGF-C, Ang-1, Ang-2, or Tie-2 (Figure 6). We speculate that there is little functional importance that can be attached to these results in relation to EC survival, proliferation, and migration because ECs possess relatively low levels of VEGF and relatively abundant levels of VEGFR1 and VEGFR2.

We found that RMST knockdown was associated with significant downregulation of cyclins D1, E1, and A2 but had no effect on CDK1 and CDK2 (Figure 6). Cyclins D1, E1, and A2 form complexes with several CDKs, including CDK1 and CDK2, to promote cell proliferation by regulating G0-G1 and G1-S transitions (Ding et al., 2011; Bendris et al., 2012). This allows cells to enter the S phase of the cell cycle. EC proliferation decreased because cyclins D1, E1, and A2 were downregulated by RMST knockdown.

The exact mechanisms through which RMST promotes EC survival, proliferation, and migration were not addressed in this study. We speculate that several are involved. First, it is possible that RMST directly interacts with specific transcription factors to promote the transcription of genes involved in survival, proliferation, and migration. RMST works this way in neurons, where it promotes neurogenic differentiation of stem cells by interacting with the transcription factor SOX2 to co-regulate a large pool of downstream genes that are essential for neurogenesis (Ng et al., 2013). Whether this is the case in ECs is unclear, as little is known about the functional roles of SOX2 in ECs, although one study has shown that SOX2 knockdown attenuates survival, proliferation, and migration in corneal ECs due to downregulation of cyclin D1 and CDK1 and upregulation of CDKN2A, a cell cycle inhibitor (Hwang et al., 2020).

Second, it also possible that RMST may interact with several RNA binding proteins. In U-251 glioblastoma cells, the pro-survival and pro-migration effects of RMST have been attributed to its direct interaction with fused in sarcoma (FUS) a DNA/RNA binding protein that is involved in mRNA transcription, maturation, and transport that promotes cell migration and proliferation (Wang et al., 2016; Liu et al., 2020). RMST also directly interacts with heterogenous nuclear ribonucleoprotein A2/B1 (hnRNPA2/B1) and heterogenous nuclear ribonucleoprotein K (hnRNPK; Ng et al., 2013; Sun et al., 2019). These ubiquitously expressed RNA binding proteins are members of the hnRNP family and are involved in mRNA splicing, post-transcriptional regulation, nuclear-cytoplasmic transport, and chromosomal stability (Wu et al., 2018). In cancer cells, hnRNPA2/B1 plays critical roles in metabolism, migration, invasion, proliferation, and cell cycle progression by inhibiting the expression of CDK inhibitors such as P21 and P27 (Shi et al., 2018).

Third, it is also possible that RMST regulates EC gene expression through DNA methylation. In MCF-7 breast cancer cells, it has been shown that RMST regulates DNA methylation through stabilization of DNA methyltransferase 3 (DNMT3; Peng et al., 2020). RMST stabilizes DNMT3 mRNA by promoting interactions between DNMT3 3’UTR and the RNA binding protein HuR; RMST knockdown suppresses DNA methylation, and upregulates methylation-regulated genes.

Fourth, it has been established that lncRNAs can act in a sponge-like manner to bind and sequester miRNAs away from their mRNA targets. RMST has recently been shown to indirectly regulate the expressions of VCAM1 in brain ECs and SEM3A in neurons by sponging miR-204-5p and miR-377, respectively, which directly target them (Yin et al., 2021; Zhao et al., 2021). To evaluate whether RMST sponges other miRNAs that negatively regulate EC survival, migration, and proliferation, we utilized a database (DIANA-lncBase v2) of experimentally supported miRNA recognition elements (MREs) on RMST (Paraskevopoulou et al., 2015). This analysis revealed that RMST has MREs for miR-27a, miR-27b, miR-33a, 132-3p, miR-139-5p, and miR-212-3p. These miRNAs have negative effects on cell survival, proliferation, and migration through direct and indirect targeting of genes involved in their regulation. For example, miR-27a directly targets vascular endothelial (VE) cadherin, an essential adhesion molecule located at the EC adherens junction that plays critical roles in EC barrier function and survival (Young et al., 2013). Furthermore, miR-27b inhibits cell proliferation and migration by targeting cyclin A2 and Smad7 (Wang et al., 2014; Rong et al., 2018), while miR-33a and miR-139-5p inhibit proliferation by targeting cyclin D1 (Sun et al., 2018; Li et al., 2020). In brain ECs, miR-33a inhibits proliferation, migration, and angiogenesis (Zhang et al., 2020). miR-132-3p and miR-212-3p target Yes-associated protein 1 (YAP1) by directly binding to its 3’UTR region to cause the downregulation of cyclin D1, which operates downstream from YAP1 (Kim et al., 2017). These diverse findings raise the possibility that RMST promotes EC survival, proliferation, and migration *via* the sponging of specific sets of miRNAs. Future studies should be dedicated to exploring this possibility.

## Conclusion

Our results indicate that several isoforms of the lncRNA RMST are expressed in human ECs and that their expressions are significantly upregulated during EC differentiation and in response to acute hypoxia. Our study also reveals that RMST promotes the pro-survival effects of the angiogenic factors VEGF and Ang-1 and enhances EC migration and proliferation. Additional studies are required to identify the exact mechanisms through which RMST regulates EC survival, proliferation, and migration.

## Data Availability Statement

The original contributions presented in the study are included in the article/**Supplementary Material**, and further inquiries can be directed to the corresponding author.

## Ethics Statement

The studies involving human participants were reviewed and approved by Ethics committee of McGill University Health Centre. The patients/participants provided their written informed consent to participate in this study.

## Author Contributions

MA: conceptualization, methodology, software, data curation, and writing – original draft. DM: methodology, software, and data curation. SH: conceptualization, software, writing – original draft, writing-review and editing, and supervision. All authors contributed to the article and approved the submitted version.

## Funding

This research was funded by Canadian Institutes of Health Research.

## Conflict of Interest

The authors declare that the research was conducted in the absence of any commercial or financial relationships that could be construed as a potential conflict of interest.

## Publisher’s Note

All claims expressed in this article are solely those of the authors and do not necessarily represent those of their affiliated organizations, or those of the publisher, the editors and the reviewers. Any product that may be evaluated in this article, or claim that may be made by its manufacturer, is not guaranteed or endorsed by the publisher.
