# Supplementary material for: Long Non-coding RNA Rhabdomyosarcoma 2-Associated Transcript Regulates Angiogenesis in Endothelial Cells
Source: Front Physiol. 2021 Oct 21;12:729157. doi: 10.3389/fphys.2021.729157 (PMC8567064; doi:10.3389/fphys.2021.729157)
Supplement: Supplementary file 1 [file Data_Sheet_1.PDF]

Supplementary Table 1: List of qPCR primers

Gene	Sequence	Accession #	Expected size (nucleotide)	Specificity
18S	F-GAGGCCATGATTAAGAGGGATG<br>R-AAACTCCGACTTTCGTTCTTGG	NC_000024	119	18S ribosomal RNA pseudogene
GAPDH	F-AAGAAGGTGGTGAAGCAGGCG<br>R- ACCAGGAAATGAGCTTGACAA	NM_002946	166	GAPDH
β-ACTIN	F: AGAAAATCTGGCACCACACC<br>R: GGGGTGTTGAAGGTCTCAAA	NM_001101	123	B-Actin
Ang-1	F- GCAGAGAGATGCTCCACACG<br>R- TATCTGGGCCATCTCCGACT	NM_001146	145	Ang-1
Ang-2	F- ATAAGCAGCATCAGCCAACCA<br>R- CATTCCGTTCAAGTTGGAAGGA	NM_001147	136	Ang-2
Tie-2	F- GGAAGGTGCCATGGACTTGA<br>R- TGACGCATCTTCATGGTTCG	NM_000459	199	Tie-2
VEGF	F- CTACCTCCACCATGCCAAGT<br>R- CACACAGGATGGCTTGAAGA	NM_001025366	281	VEGF
VEGF-C	F- TGTACAAGTGTCAGCTAAGG<br>R- CCACATCTATACACACCTCC	NM_005429	187	VEGF-C
VEGF-R1	F- CACTGGGCAGCAGACAAATC	NM_002019	109	Flt-1

	R- TCACACCTTGCTTCGGAATG			
VEGF-R2	F- GGCTTTGGCCCAATAATCAG<br>R- ATGACCGAGGCCAAGTCAGT	NM_02253	208	KDR
Cyclin D1	F- CTGTGCTGCGAAGTGGAAAC<br>R- CCTCCTCCTCGCACTTCTGT	NM-053056	214	CyclinD1
Cyclin A2	F- GCTCCAAGAGGACCAGGAGA<br>R- AGGAGGAACGGTGACATGCT	A32139	192	Cyclin A2
Cyclin E1	F- GGGACACCATGAAGGAGGAC<br>R- GATGCTCCCCAACCTGTCTC	X95406	105	Cyclin E1
CDK1	F- TGGGGTCAGCTCGTTACTCA<br>R- TTATTGGGAGTGCCCAAAGC	NM_001786	148	CDK1
CDK2	F- AAGATCGGAGAGGGCACGTA<br>R- ACTGGGCACACCCTCAGTCT	NM_001798	114	CDK2
RMST-201	F: TCCGTTTGGAATAAGGCAGA<br>R: R: CAAGCTTGGTGAGCCAAACA		164	RMST-201
RMST-202	F: ACCACCATCCCAACAAAAGC<br>R: R: CGGTGGTTGGCACTGAACTA	NR_024037	150	RMSR-202
RMST-203	F: ACCTTGGATCTAAGCAGCACCT<br>R: GATCCAACCTAGGCATTTAGGC		99	RMST-203, RMST-209, RMST-210

RMST-204	F: GGCTGATGCTTCTTGCCTGT<br>R: GGACCTTCAAGGGAGTAAACTGG	115	RMST-204
RMST-205	F: GACATGCCCAAAGAAGTCTGC<br>R: GGAACACCATCTGCCTTTGG	180	RMST-205
RMST-206	F: AGACAGCTGGTGACGCATGT<br>R: CCTCTCCTCAGCCATCGACT	116	RMST-206
RMST-207	F: CGCTGAATATCTTCAGGAA<br>R: TGACATCCTCCTCGAATCA	36	RMST-207
RMST-208	F: ATTGCTGGGGTTGCAAAGAG<br>R: TGGTTTAGTTTCCCCGTCCTT	186	RMST-208
RMST-209	F: GGAGCCTGACTGTGAGAACTG<br>R: TGGATTCCGAAACCCAGAAG	300	RMST-209
RMST-210	F: CACCCACTCTCAACACTGTGC<br>R: CGGATCCTGTTCCAGCAACT	109	RMST-210

Supplementary Table 2: List of lncRNAs which were significantly upregulated by 24 hours of hypoxia as detected by RT^2 lncRNA PCR Array Human lncFinder (Qiagen Inc.).

Symbol	Name	RefSeq Number	Fold change from normoxia
CCAT2	Colon cancer associated transcript 2	NR_109834	13.2± 2.31
GAS5	Growth arrest-specific 5	NR_002578	1.58± 0.10
H19	H19, imprinted maternally expressed transcript	NR_002196	4.05± 0.52
HOTAIR	Hox transcript antisense RNA	NR_003716	5.20± 0.95
Malat1	Metastasis associated lung Adenocarcinoma 1	NR_02819	2.1± 0.33
RMST	Rhabdomyosarcoma 2 associated transcript	NR_024037	19.4± 3.24
SOX2-OT	SOX2 overlapping transcript	ENST00000410534	8.31± 0.91

Values are means ± SEM. N=3 per group (normoxia and hypoxia)

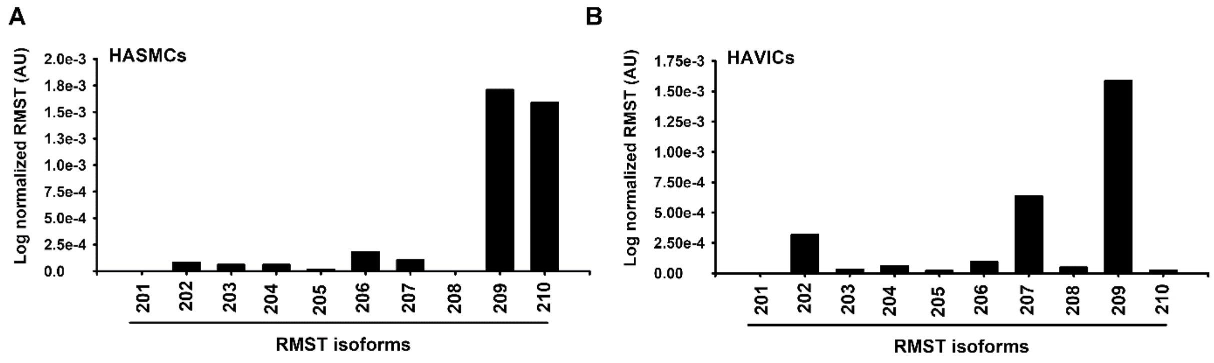

Supplementary Figure 1: Relative expression of RMST isoforms in normoxic human aortic skeletal muscle smooth muscle cells (HASMCs) and human aortic valvular interstitial cells (HAVICs). Values are mean of three different measurements.

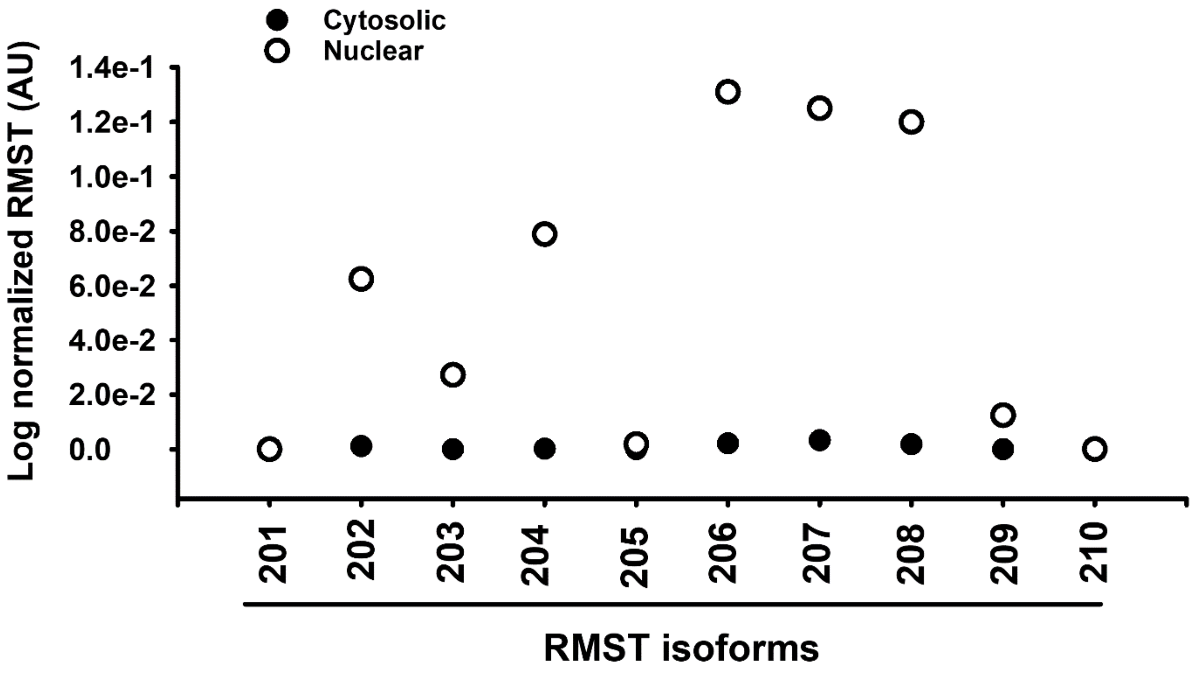

Supplementary Figure 2: Relative expression of RMST isoforms in the nuclear and cytosolic fractions of HUVECs. Values are mean of three different preparations.

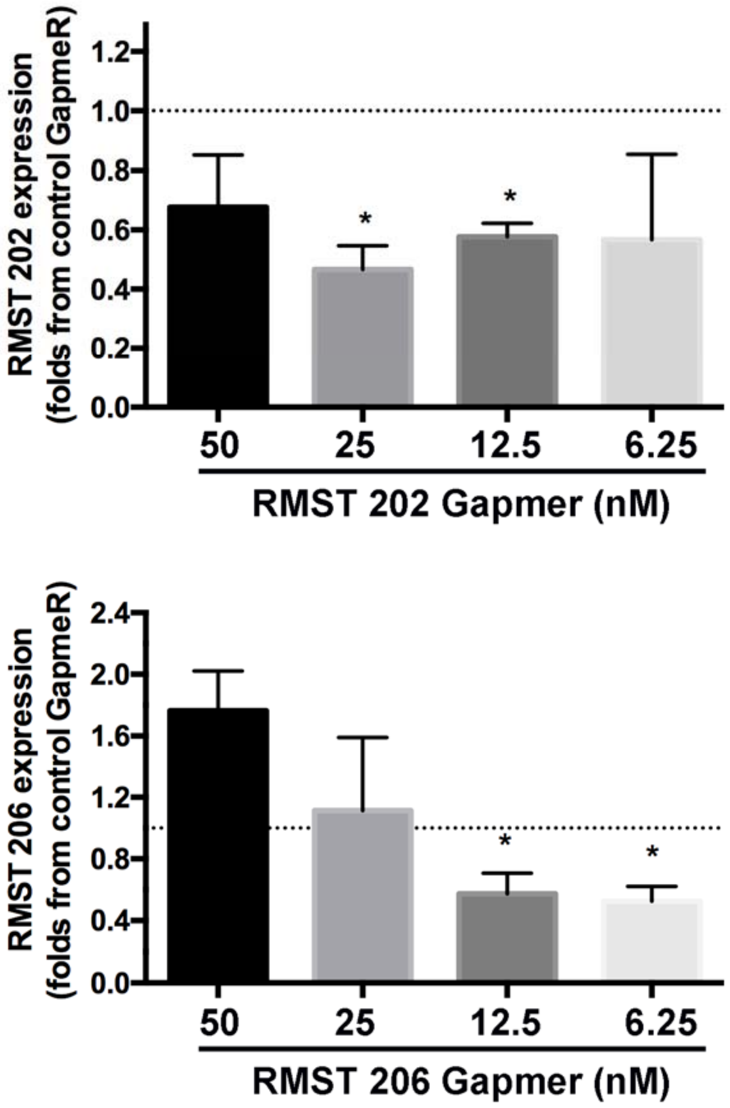

Supplementary Figure 3: Effects of transfection with different concentrations of RMST-202 and RMST-206 GapmeR on their corresponding lncRNA expressions. * $p<0.05$, compared to control GapmeR. Values are mean of three different preparations.
